# Improving glucose and lipids metabolism: drug development based on bile acid related targets

**DOI:** 10.15698/cst2021.01.239

**Published:** 2021-01-05

**Authors:** Hanchen Shen, Lili Ding, Mehdi Baig, Jingyan Tian, Yang Wang, Wendong Huang

**Affiliations:** 1 School of Pharmacy, Fudan University, Shanghai 201203, China.; 2 Shanghai Key Laboratory of Complex Prescriptions and MOE Key Laboratory for Standardization of Chinese Medicines, Institute of Chinese Materia Medica, Shanghai University of Traditional Chinese Medicine, Shanghai, 201203, China.; 3 Department of Diabetes Complications and Metabolism, Institute of Diabetes and Metabolism Research Center, Beckman Research Institute, City of Hope National Medical Center, 1500 E. Duarte Road, Duarte, CA 91010, USA.; 4 Shanghai Institute of Endocrine and Metabolic Diseases, Ruijin Hospital, Shanghai Jiao Tong University School of Medicine, Shanghai 200025, China.

**Keywords:** bile acid, FXR, TGR5, GPBAR1, bile salt hydrolase, CYP8B1

## Abstract

Bariatric surgery is one of the most effective treatment options for severe obesity and its comorbidities. However, it is a major surgery that poses several side effects and risks which impede its clinical use. Therefore, it is urgent to develop alternative safer pharmacological approaches to mimic bariatric surgery. Recent studies suggest that bile acids are key players in mediating the metabolic benefits of bariatric surgery. Bile acids can function as signaling molecules by targeting bile acid nuclear receptors and membrane receptors, like FXR and TGR5 respectively. In addition, the composition of bile acids is regulated by either the hepatic sterol enzymes such as CYP8B1 or the gut microbiome. These bile acid related targets all play important roles in regulating metabolism. Drug development based on these targets could provide new hope for patients without the risks of surgery and at a lower cost. In this review, we summarize the most updated progress on bile acid related targets and development of small molecules as drug candidates based on these targets.

## INTRODUCTION

**Figure 1:**
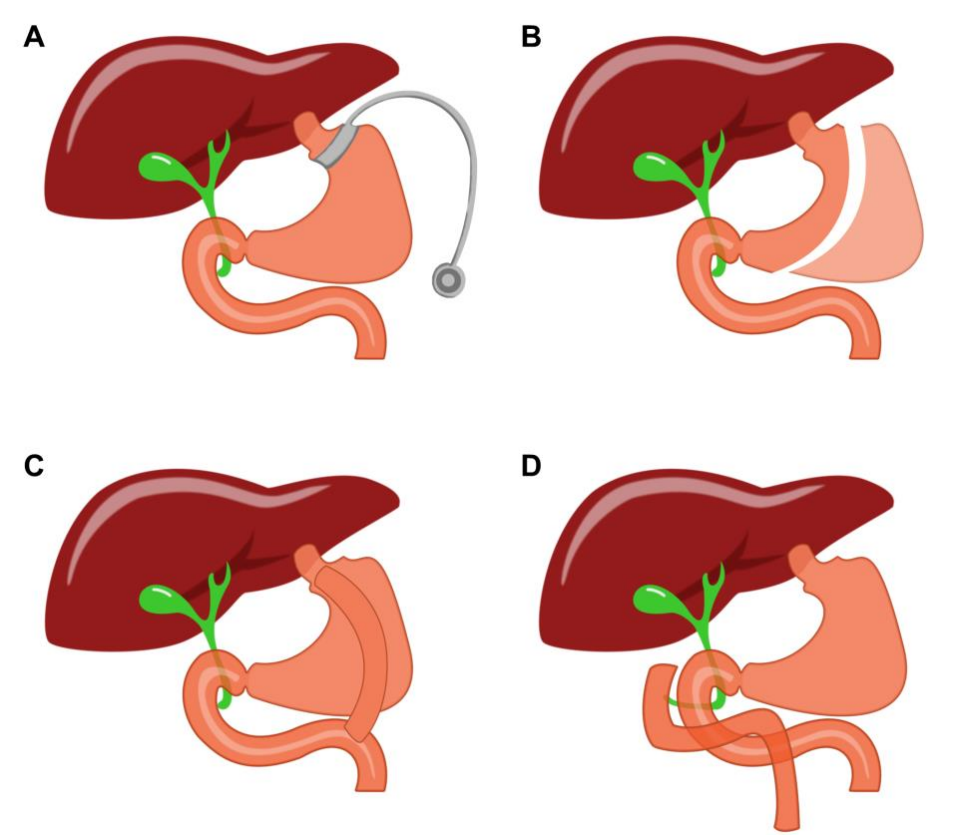
FIGURE 1: Contemporary procedures for metabolic surgery. **(A)** adjustable gastric banding (AGB); **(B)** vertical sleeve gastrectomy (VSG); **(C)** Roux-en-Y gastric bypass (RYGB); and **(D)** biliopancreatic diversion with duodenal switch (BPDDS).

The incidence of obesity continues to rise rapidly in industrialized countries, including the US. Obesity is defined by an excessive amount of body fat that impairs health. While mortality of patients is not directly caused by obesity itself, patients who develop comorbidities are likely to have a much higher mortality rate. These comorbidities include cardiovascular disease, diabetes mellitus, kidney related diseases, certain cancers, anxiety, depression, and degenerative joint disorders [1]. Bariatric surgeries, including Roux-en-Y gastric bypass (RYGB), vertical sleeve gastrectomy (VSG), adjustable gastric banding (AGB) and biliopancreatic diversion with duodenal switch (BPDDS), are the most effective therapies for the treatment of severe obesity (**Fig. 1**) [2]. It can be done for sickly obese patients who have no response to pharmacological or behavioral treatment [3]. Nowadays, the most common bariatric surgeries are RYGB and VSG, both of which change people's digestive system to reduce weight and improve glucose tolerance [4]. Despite its efficiency, the clinical use of bariatric surgery is still impeded by cardiovascular risk and other serious complications [2]. For this reason, there is an urgent need to find less-invasive therapeutic approaches to mimic metabolic surgery.

Traditionally, bariatric surgeries were perceived to show their efficacy by manipulating the anatomy of the gastrointestinal tract, and consequently limiting nutrient absorption. Different surgeries like RYGB and VSG have been able to target weight loss in their own ways. It can be noted that these differing surgical techniques are able to achieve similar metabolic results. This would suggest that altering the anatomy of the stomach and intestine is not solely responsible for the weight loss. Rather, there are supplementary physiological phenomena involved, other than simply restricting the amount of food absorbed by the body [5, 6]. Recent studies showed that the primary mechanism could be because of the alternation in secretion and activity of hormones and neurotransmitters [4, 7–9]. This molecular component, rather than the mechanical restriction, can explain the beneficial impact of bariatric surgeries. Developing drugs or related therapies based on the molecular mechanism is thus a promising way to treat obese patients without surgical trauma.

Among those mechanisms, bile acids play a central role. Several studies have focused on the elevated serum bile acid levels after bariatric surgeries, which led to better glucose and lipids metabolism [10, 11]. Bile acids are detergent molecules, and are required for absorption of fats, steroids, and lipid-soluble vitamins in the intestine [12]. They are also signaling molecules that activate nuclear and membrane bile acid receptors, through which bile acids can modulate lipid and glucose metabolism [13]. Bile acids can also behave as antibacterial agents in the gut. They are involved in a complex interaction with the gut microbiota, and are essential for normal gastrointestinal function [14]. In spite of the fact that they are vital for human health, bile acids are highly toxic when they accumulate in the liver and other peripheral tissues [15]. Due to the prominent physiological effects of bile acids, developing drugs based on bile acid related targets are actively pursued by pharmaceutical companies.

## NUCLEAR AND MEMBRANE BILE ACID RECEPTORS

In addition to digestive function, bile acids are also versatile signaling molecules that activate multiple signaling pathways [13]. The main functions of bile acids depend on the activation of two receptors: nuclear farnesoid X receptors (FXR) and membrane G-protein-coupled Takeda G-protein-coupled receptor 5 (TGR5) [16]. These two receptors are distributed differently in tissues and have a wide range of physiological functions. Thus, they became promising pharmacological targets in treating diseases such as primary biliary cholangitis (PBC), primary sclerosing cholangitis (PSC), cholestatic diseases, and other metabolic diseases [17].

### Nuclear farnesoid X receptor, FXR

FXR is mainly expressed in various tissues including the liver, intestine, kidney, and adrenal gland, and it plays crucial roles in the regulation of bile acid synthesis, secretion, and transport [18]. FXR is important for lipid and glucose metabolism; the activation of FXR has shown beneficial effects on various metabolic diseases such as nonalcoholic fatty liver disease (NAFLD), type 2 diabetes, dyslipidemia, and obesity [19]. Ryan *et al.* demonstrated that FXR is required to for the metabolic improvements of VSG [8]. These results indicate that FXR agonism plays an essential role in “bariatric-mimetic” technology.

**Figure 2:**
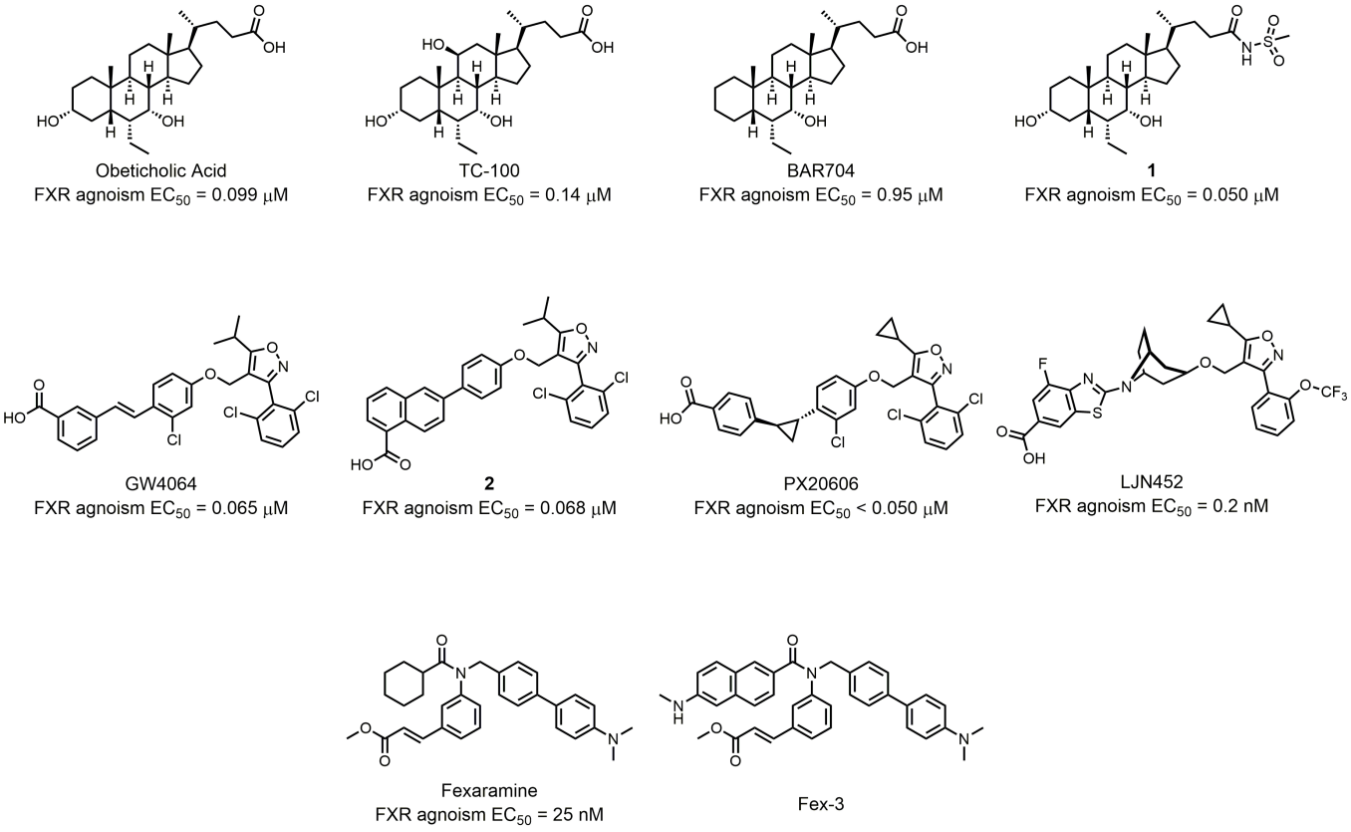
FIGURE 2: Semisynthetic bile acid derivatives as FXR agonists and synthetic FXR nonsteroidal.

As a nuclear receptor of bile acids, FXR can be activated by several bile acids at physiological concentrations. CDCA (chenodesoxycholic acid) is the most potent ligand among all the bile acids in human, with EC_50_ values ranging between 2-5 μM[20]. Because of its high FXR activating potency, the chemical manipulation of the CDCA scaffold offers many compounds with improved potency, efficacy, and metabolic stability. Pellicciari *et al.* synthesized a series of 6α-alkyl-substituted analogues of CDCA and evaluated their binding potency to FXR [21]. 6α-ethyl-chenodeoxycholic acid (6-ECDCA) was shown to be a very potent and selective FXR agonist (**Fig. 2**). With FXR agonism EC_50_ = 0.99 μM, 6-ECDCA showed anticholeretic activity in an *in vivo* rat model of cholestasis. 6-ECDCA, also known as obeticholic acid or INT-747, is now the most promising FXR agonist for clinical use. It has been approved for the treatment of ursodeoxycholic acid (UDCA) resistant patients in PBC [22]. Additionally, 6-ECDCA is now advancing through Phase III clinical trials in nonalcoholic steatohepatitis (NASH) patients (NCT02548351). Further research was conducted to improve the potency, and physicochemical properties of CDCA derivates. Pellicciari *et al.* state that modified hydroxyl groups on steroidal nucleus can affect the properties and behavior of different bile acids [23]. Based on docking studies of hydroxyl derivatives of 6-ECDCA, they designed and synthesized a series of semisynthetic bile acid derivatives. Of those, TC-100 not only boasted the highest FXR agonist activity with an EC_50_ of 0.14 μM, but also had improved physicochemical profiles compared to that of 6-ECDCA (**Fig. 2**). However, many FXR agonists, including 6-ECDCA, also have TGR5 agonist activity which can cause adverse effects like pruritus. To solve this problem, Sepe *et al.* conducted chemical modifications on the C3-hydroxyl group of the steroidal core? [24]. This strategy generated a potent FXR agonist BAR704 (EC_50_ = 0.95 μM) with inhibitory activity against TGR5 (**Fig. 2**). Xiao *et al.* further found that modification on the side chains can change the properties of bile acid derivates. They substituted carboxylic tail with amide groups generating compound **1** with good efficacy, FXR selectivity, and pharmacokinetic properties compared to 6-ECDCA (**Fig. 2**) [25].

The FXR agonists discussed above all contain a steroidal core?. However, the steroidal bile acid-like chemical structure suffer some limitations such as poor aqueous solubility, poor bioavailability, unattractive pharmacokinetic profile, and low FXR selectivity [26]. For these reasons, research of synthetic nonsteroidal FXR agonists has been gaining attention in recent years. Maloney *et al.* utilized a cell free ligand-sensing assay to identify an isoxazole compound as the lead compound from a combinatorial library of 9,900 stilbene carboxylic acids [27]. Further structure-activity-relationship (SAR) studies helped researchers to find GW4064, the first high-affinity nonsteroidal FXR agonist (**Fig. 2**). Despite the high potency, the clinical use of GW4064 was limited because of toxicity, poor pharmacokinetic properties, and stilbene-mediated photo-instability. Akwabi-Ameyaw *et al.* designed and synthesized compound **2** (EC_50_ = 0.068 μM), a stilbene replacement of GW4064 (EC_50_ = 0.065 μM; **Fig. 2**) [28]. Compound **2** exhibited comparable efficacy and selectivity to GW4064, and could reduce the severity of cholestasis in the a-naphthyl-isothiocyanate (ANIT) acute cholestatic rat model. However, the oral bioavailability of compound **2** in rodents was poor. Kinzel *et al.* studied the SAR of terminal carboxylic acid-bearing aryl or heteroaryl moiety and linker group of GW4064 derivates [29]. Through the SAR study, they found that PX20606 was a potent FXR agonist (EC_50_ < 0.1 μM) with improved aqueous solubility and metabolic stability (**Fig. 2**). PX20606 demonstrated beneficial effects in two animal models of prehepatic and intrahepatic portal hypertension [30]. To date, two phase I studies of PX20606 have been completed (NCT01998659, NCT01998672), showing potential for further clinical use. Tullu *et al.* conducted a thorough SAR study of isoxazole derivates, paying attention to potency and pharmacokinetic properties at the same time [31]. By introducing a bicyclic nortropine-substituted benzothiazole carboxylic acid moiety, the researchers synthesized LJN452 with an EC_50_ of 0.2 nM (**Fig. 2**). LJN452 was found to be generally safe and well-tolerated at pharmacologically active doses in healthy volunteers. Now LJN452 has completed two phase II studies for the treatment of patients with PBC (NCT02516605) and NASH (NCT02855164). Besides isoxazole derivates, many compounds with other structures can also act as starting compound in the development of synthetic nonsteroidal FXR agonists. Downes *et al.* screened for hit compounds from a combinatorial library of ≈ 10,000 benzopyran-based compounds; further modification and screening generated the potent FXR agonist fexaramine (EC_50_ = 25 nM) (**Fig. 2**) [32]. Fexaramine was poorly absorbed into circulation when delivered orally, and its intestinal-restricted property showed the possibility of a promising and safer treatment [33]. For this reason, Wang *et al.* synthesized Fex-3, which targets FXR in the ileum and has a better selectivity [34].

**Figure 3:**
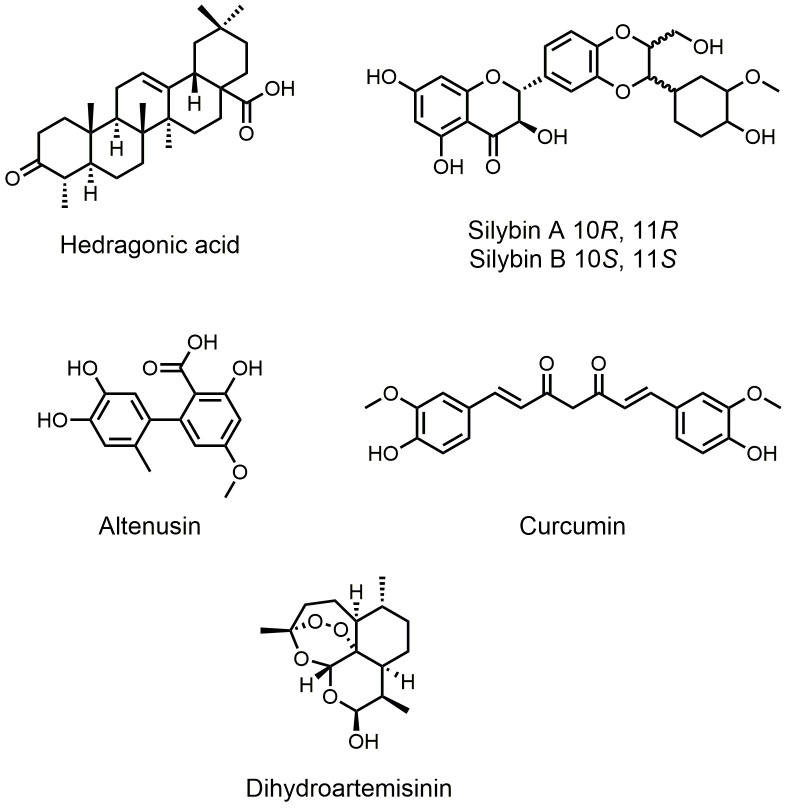
FIGURE 3. Natural FXR agonists.

Besides semisynthetic bile acid derivatives and synthetic nonsteroidal FXR agonists, many natural products also show FXR agonist activity. Interestingly, these natural products have greater structural diversity. Studies focusing on natural FXR agonists helped researchers gain more insights into the structures of FXR ligands and can lead to innovative drug design and synthesis. Lu *et al.* showed that hedragonic acid, isolated from the stem and root of *Celastrus orbiculatus* Thunb, is a selective FXR agonist with anti-inflammatory and liver protection activity (**Fig. 3**) [35]. Although hedragonic acid belongs to oleanane-type triterpene, whose structure is similar to the steroidal core of bile acids, FXR has a unique binding mode with it. Hedragonic acid occupies a novel binding pocket in FXR, which is different from the classic binding position. *In vivo* experiments demonstrated outstanding therapeutic effects of hedragonic acid in liver diseases, suggesting its potential for clinical use. Curcumin exhibits a wide range of biological activities, including anticancer, antimicrobial, anti-inflammatory, and antioxidation activities (**Fig. 3**) [36]. Danning tablet, a Chinese patent medicine preparation, has been clinically used to treat human liver and gallbladder diseases. Yang *et al.* found that curcumin, one major compound derived from danning tablet, exerted the therapeutic effect [37]. Binding to FXR, curcumin could reduce hepatic bile acids accumulation, normalized the imbalance of bile acid homeostasis, and reduced inflammatory responses. Through molecular docking experiments, the researchers found that curcumin could theoretically bind to the FXR protein at the same site as GW4064, suggesting more structure flexibility at this binding site. Silymarin, consisting of seven flavonolignans, is a natural herbal extraction from the fruit and seeds of the *Silybum marianum* (**Fig. 3**). Silymarin displays a wide range of bioactivities, including antioxidant, anti-inflammatory, anti-proliferative, and immunomodulatory effects [38]. Gu *et al.* reported that silymarin could generate benefits in insulin resistance, hyperlipidemia, and inflammation progress in Diet-induced obesity (DIO) mice model, and silybin A and B are major constituent of silymarin, showing higher activity [39]. Molecular docking showed that the 6-ECDCA binding site of FXR could be validated as the binding pocket for silybin, suggesting a structural basis for silybin activity. Altenusin is a nonsteroidal fungal metabolite isolated from *Penicillium sp* (**Fig. 3**) [40]. Zheng *et al.* used a GAL4-FXR-LBD chimeric receptor to screen for altenusin as a novel FXR agonist (EC_50_ = 3.4 ± 0.2 μM) [41]. Evaluation in the same luciferase reported assay showed that this compound has a potency and efficacy similar to those of CDCA (EC_50_ = 3.8 ± 0.3 μM). *In vivo* experiments demonstrated that administration of altenusin to high-fat diet (HFD)-induced obese mice produced metabolic benefits. Molecular docking analysis proved that altenusin can bind to FXR at the FXR ligand-binding domain (LBD), which is the binding site between CDCA and FXR. Artemisinin is a δ-sesquiterpene lactone endoperoxide isolated and identified from *Artemisia annua* L by more than 500 Chinese scientists [42]. Dihydroartemisinin (DHA) is a semisynthetic derivative of artemisinin, and it was identified as a FXR ligand (**Fig. 3**) [43]. DHA showed desirable therapeutic action on alcohol-induced liver injury, inflammation, and steatosis in rat via an FXR-dependent mechanism.

### Membrane G-protein-coupled Takeda G-protein-coupled receptor TGR5

TGR5, also called G-protein bile acid-activated receptor (GPBAR1), is widely expressed in many tissues, including liver, gallbladder, brown adipose tissue and the intestine [44]. In the intestine, TGR5 promotes the secretion of glucagon-like peptide 1 (GLP-1) and anorectic hormone peptide YY (PYY) by L-cells [45]. GLP-1 improves glucose metabolism by inducing glucose-dependent stimulation of insulin secretion, suppressing glucagon secretion, and stimulating the proliferation and differentiation of insulin-secreting beta cells. PYY, on the other hand, has been shown to reduce appetite, and is helpful for overweight and obese patients. TGR5 activation also increases energy expenditure in tissues like brown adipose tissue and human skeletal muscles [46]. TGR5 activation converts inactive thyroxine (T4) into the active iodothyronine (T3) form by triggering the thyroid hormone-activating enzyme type 2 iodothyronine deiodinase (D2). TGR5 also plays important roles in cell signaling [47]. When activated by bile acids with different affinities, TGR5 can upregulate levels of cyclic adenosine monophosphate (cAMP) by interacting with G protein α_S_ (Gα_S_). Several downstream signaling pathways could thus be stimulated, thereby performing a wide range of physiological functions. Chen *et al.* reported that when TGR5 was activated by oleanolic acid (OA), the expression of microRNA-26a (miR-26a) in macrophages was strongly increased [48]. MiR-26a can bring beneficial effects in regulating insulin sensitivity and metabolism of glucose and lipids, and is a potential target for the treatment of type 2 diabetes [49]. These results showed a miRNA-mediated mechanism of metabolic improvement through TGR5 activation. Ding *et al.* reported that the expression of TGR5 increased significantly after VSG in a mouse model [4]. With increased levels of bile acids caused by VSG, TGR5 signaling in the ileum and brown adipose tissues were significantly enhanced, showing improved glucose control and increased energy expenditure. It is thus possible for TGR5 agonism by small molecules to provide metabolic benefits to mimic bariatric surgery.

**Figure 4:**
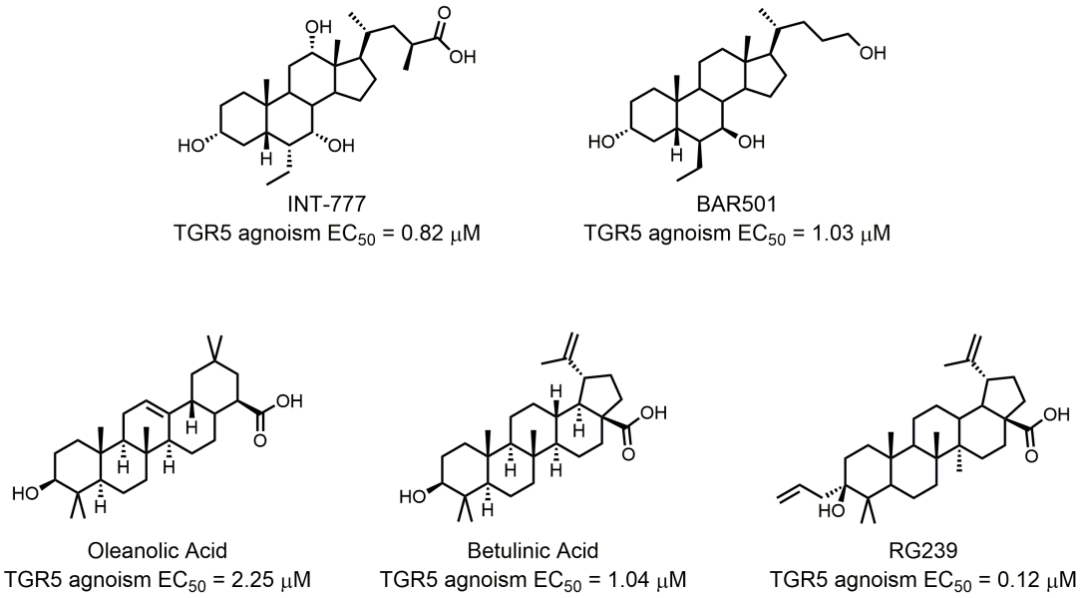
FIGURE 4: Semisynthetic TGR5 agonists.

The endogenous natural agonists of TGR5 are bile acids, with lithocholic acid (LCA) and taurolithocholic acid (TLCA) being the most potent endogenous agonists [50]. The EC_50_ of LCA and TLCA are 0.53 μM and 0.33 μM, respectively. Pellicciari *et al.* found that 23(*S*)-methyl substitution on a CDCA side chain afforded TGR5 selectivity to bile acids [51]. According to the ideal pharmacokinetic properties and favorable metabolic profiles of cholic acid? (CA), they generated 6R-ethyl-23(*S*)methylcholic acid (INT-777) as a novel potent and selective TGR5 agonist with enhanced *in vivo* activity (EC_50_ = 0.82 μM) (**Fig. 4**). INT-777 efficiently increased GLP-1 secretion *in vivo*, and it could thus increase energy expenditure, reduce hepatic steatosis, and resulted in a significant reduction in weight gain and adiposity [52]. Kumar *et al.* recently demonstrated that administration of INT-777 to db/db mice improved pancreatic β-cell proliferation, insulin synthesis, and insulin release, showing its potential in treating diabetes mellitus and obesity [53]. Festa *et al.* further conducted detailed SAR studies, discovering the relationship between the ligand selectivity and the modification of the side chain and the cholane scaffold [54]. This study led to the identification of BAR501, an alcohol derivative of UDCA with potent and selective TGR5 activity (EC_50_ = 1.03 μM; **Fig. 4**). BAR501 could reverse insulin resistance, ameliorate liver histology, promote browning of epididymal white adipose tissue (epWAT), and increase energy expenditure in a rodent model of NASH [55]. Besides these semisynthetic bile acids, other natural products also demonstrate TGR5 agonist activity. OA is an active triterpene from *Olea europaea* and the first and best studied example of a natural TGR5 ligand (**Fig. 4**). It has an TGR5 agonist activity comparable with that of LCA [56]. Kumar *et al.* found that TGR5 activation in pancreatic β cells by OA could improve glucose tolerance and insulin release, highlighting the importance of targeting TGR5 in the control of glucose homeostasis [57]. Betulinic acid (BTA) is another identified active triterpene able to activate TGR5 (**Fig. 4**). It was found by screening a collection of natural triterpenoids [58]. In mice model fed a HFD, BTA increased insulin and leptin levels and decrease blood glucose and lipid levels [59]. However, whether the beneficial effects were related to TGR5 remains unknown. Structural modifications of the C-3 alcohol, the C-17 carboxylic acid, and the C-20 alkene on the BTA scaffold resulted in the finding of RG-239 (**Fig. 4**) [58]. RG-239 boasted a largely increased potency to activate TGR5 (EC_50_ = 0.12 μM) compared to that of BTA (EC_50_ = 1.04 μM) and OA (EC_50_ = 2.25 μM). Despite its high potency, however, RG-239 had a poor result tested for its antidiabetic properties *in vivo*. This result underlines the importance of suitable pharmacokinetic properties.

**Figure 5:**
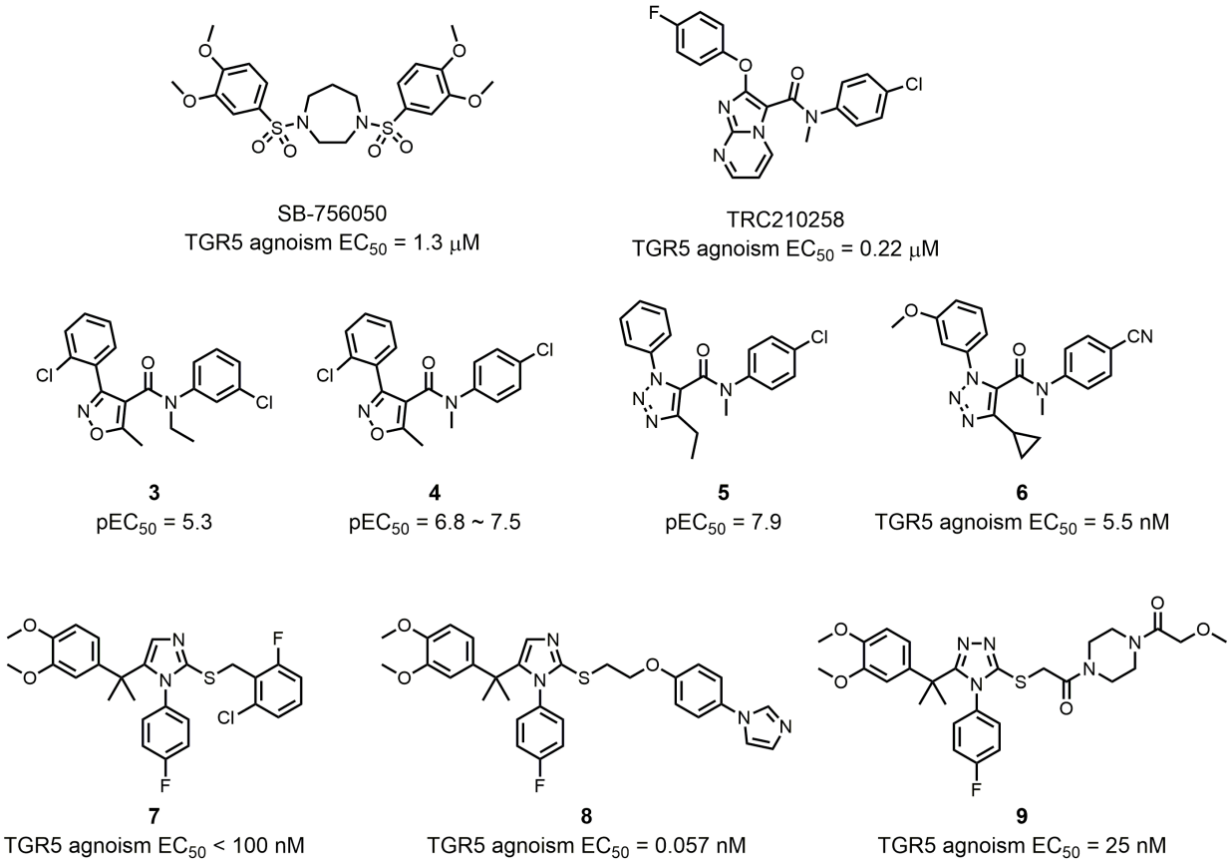
FIGURE 5: Synthetic nonsteroidal TGR5 agonists.

In order to avoid potential side effects from the steroidal structure and to explore the structural diversity of TGR5 agonists, several examples of nonsteroidal compounds have been reported as TGR5 agonists by various pharmaceutical companies and research groups. Szewczyk *et al.* disclosed a series of bis-phenyl sulfonamide TGR5 agonists in 2007 [60]. Among those agonists, SB-756050 is a selective TGR5 agonist (EC_50_ = 1.3 μM), and its use in humans has been investigated and published (NCT00733577; **Fig. 5**) [61]. Although chronic enteral administration of SB-756050 in diabetic rats could increase plasma GLP-1 levels and improve glucose metabolism [60], the same effects could hardly be shown in humans because the pharmacodynamic effects of SB-756050 in humans were complex and inconsistent [61]. This result indicates that differences exist between rat and human TGR5 targeting. TRC210258 was found by Zambad *et al.* by screening ~400 compounds for their ability to activate TGR5 *in vitro* (**Fig. 5**) [62]. In diet-induced obesity (DIO) mice and hamsters, TGR5 activation by TRC210258 could reduce cardiovascular risks by improving metabolic parameters, such as GLP-1 secretion, energy expenditure, cholesterol and glucose metabolism.

Some other compounds were identified and modified as chemical templates. Evans *et al.* employed a high-throughput screen using a BacMam transduced human osteosarcoma cell line (U2-OS) to identify isoxazole compound **3** as a TGR5 agonist with a pEC_50_ of 5.3 and 100% maximum response (**Fig. 5**) [63]. SAR optimization of the amide phenyl ring produced compound **4**, showing improved potency in the U2-OS cell assay (pEC_50_ = 6.8) and in melanophore cells (pEC_50_ = 7.5) (**Fig. 5**). In a conscious dog model, coadministration of compound **4** and glucose afforded a significant improvement in portal vein GLP-1 secretion and glucose reduction, showing its potential to be a useful therapeutic for metabolic disorders. However, high intrinsic clearance in rat liver microsomes limited further application of compound **4**. Budzik *et al.* found that replacement of the isoxazole with a 1,2,3-triazole could reduce intrinsic clearance, showing promising improvements in the *in vitro* metabolic stability profile [64]. The generated compound **5** maintain good *in vitro* potency (pEC_50_ = 7.9) (**Fig. 5**). Using compound **5** as starting point because of its favorable physicochemical properties (molecular weight = 341, elogD^14^ = 2.9, and topological polar surface area = 51) and conformational rigidity (number of rotatable bonds = 6), Futatsugi *et al.* further optimized triazole based compound **5** in order to identify orally available compounds by maximizing TGR5 potency and improving metabolic stability [65]. Although compound **6** was not the most potent TGR5 agonist in this literature, it showed the best balance of TGR5 activity and *in vitro* clearance (**Fig. 5**).

In 2010, Bollu *et al.* from Exelixis reported a series of triazole and imidazole TGR5 agonists [66]. Compound **7** showed receptor activation with EC_50_ values less than 100 nM in both the TGR5 cAMP assay and the TGR5/CRE-luciferase assay (**Fig. 5**). Agarwal *et al.* utilized Exelixis compound **7** as starting point for generating novel TGR5 agonists [67]. Molecular docking studies based on a TGR5 homology model showed that a linker with hydrogen bond acceptor and conformational flexibility could be beneficial for the interaction between small molecules and TGR5.

Further substitution on the terminal phenyl ring generated potent TGR5 agonist compound **8** (EC_50_ = 0.057 nM), which had outstanding TGR5 selectivity (**Fig. 5**). Besides potency, compound **8** also had better improved pharmacokinetic profile, rendering it an orally efficacious drug candidate. In order to expand the chemical pool with novel compounds for potential TGR5 agonist candidate, Agarwal *et al.* continued to investigate substituents on the right-hand side of the molecule, which were linked to the imidazole or triazole core with an amide linker [68]. Compound **9** with a triazole core had the highest hydrophilicity, which ensured compound **9** a low systematic exposure and minor side effects, while having an excellent TGR5 agonistic activity at the same time (**Fig. 5**). Molecular docking studies demonstrated that the tail piperazine ring and the fluoro-phenyl group of compound **9** could form hydrogen bonds and π-π stacking interactions with TGR5, explaining its high affinity to TGR5 (EC_50_ = 25 nM).

**Figure 6:**
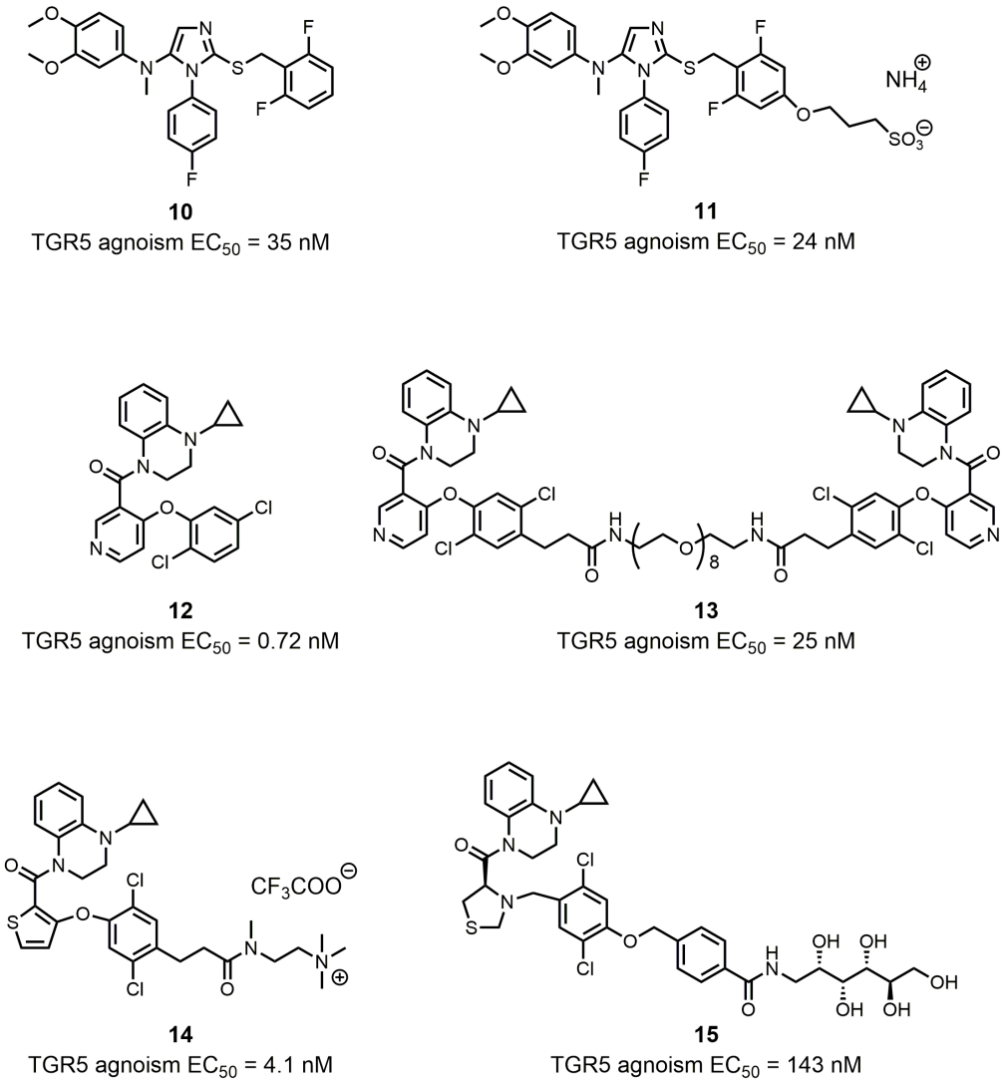
FIGURE 6: Synthetic intestinally targeted TGR5 agonists.

Activation of TGR5 has a wide range of bioactivities within different cells and tissues [47]. Systemic TGR5 agonists may trigger unwanted effects such as gallbladder swelling, itching, or cardiovascular issues [65, 69, 70]. For this reason, it was hypothesized that localized activation of TGR5 within the intestine could help minimize side effects during therapy. Different chemical modulation strategies have been utilized to produce intestinally-targeted TGR5 agonists. Lasalle *et al.* used the compound **8** derivate compound **10** as a chemical template to design and synthesize topical intestinal agonists (**Fig. 6**) [71]. Through combining a “pharmacophore” (compound **10**) bearing the pharmacological activity and a “kinetophore” (sulfonate group) controlling the pharmacokinetic properties, compound **11** had a much-reduced cellular permeability, while its activation of TGR5 remained good, which was essential for its excellent potency (**Fig. 6**). The combination of other pharmacophores and kinetophores was also reported by different groups. Compound **12** is an orally efficacious TGR5 agonist based on 4-phenoxynicotinamide (EC_50_ = 0.72 nM) (**Fig. 6**) [72]. While exposure of compound **12** to the intestine caused the secretion of GLP-1, its exposure to other tissues such as gallbladder and heart still resulted in unwanted side effects. In order to reduce the passive molecular transporting through membranes, Duan *et al.* designed and synthesized (polyethylene glycol) PEG-containing derivatives of compound **12** [73]. The PEG_8_ derivative compound **13** exhibited a permeability much lower than that of the small molecule TGR5 agonist **12** in Caco-2 permeability assays, and compound **13** showed satisfactory potency both *in vitro* (EC_50_ = 25 nM) and *in vivo* (**Fig. 6**). Compound **12** and its derivates underwent other chemical modulation. Bile acid sequestrants, as cholesterol-lowering polymer drugs, can bind to bile acids in the intestine. They are barely absorbed in the gut because of their high molecular weight and positive charge [74]. The quaternary ammonium structure plays an important role in the non-absorbed profile of bile acid sequestrants. Inspired by this, Cao *et al.* incorporated a quaternary ammonium structure with compound **12** derivates, in which the pyridine ring of **12** was replaced by a thiophene ring, to generate compound **14** (**Fig. 6**). Compound **14** was a potent TGR5 agonist (EC_50_ = 4.1 nM), and it was shown to be intestinally targeted through pharmacokinetic studies. Although the gallbladder filling effect of **14** was decreased in mice, however, this side effect was still not eliminated. Chen *et al.* tuned the performance of TGR5 agonists in the gastrointestinal lumen through systematic SAR optimization [75]. The optimized compound **15** had a similar pharmacophore as compound **12**, expect for its thiazolidine structure, and the kinetophore was D-glucamide (**Fig. 6**). Compound **15** exhibited high potency as shown by *in vitro* TGR5 agonism (EC_50_ = 143 nM), and it did not inhibit gallbladder emptying. It could stimulate robust and sustained GLP-1 secretion with minimal TGR5-mediated suppression of gallbladder emptying, which rendered **15** an ideal therapeutic method for patients with metabolic diseases such as type 2 diabetes, NASH, or inflammatory bowel disease. Also, compound **15** could be an ideal tool for elucidating the physiological responses of TGR5 agonism without systemic target engagement.

### FXR and TGR5 dual targeting agonists and other dual targeting compounds

Although many efforts have been made to develop selective FXR or TGR agonists with reduced side effects, research shows that dual agonism of FXR and TGR5 is extremely useful in the treatment of diabetes mellitus, obesity [76], NASH [55, 77], atherosclerosis [78], and even other diseases like bone loss [79]. These results demonstrate the potential of FXR/TGR5 dual-targeting agonists as a promising therapy.

**Figure 7:**
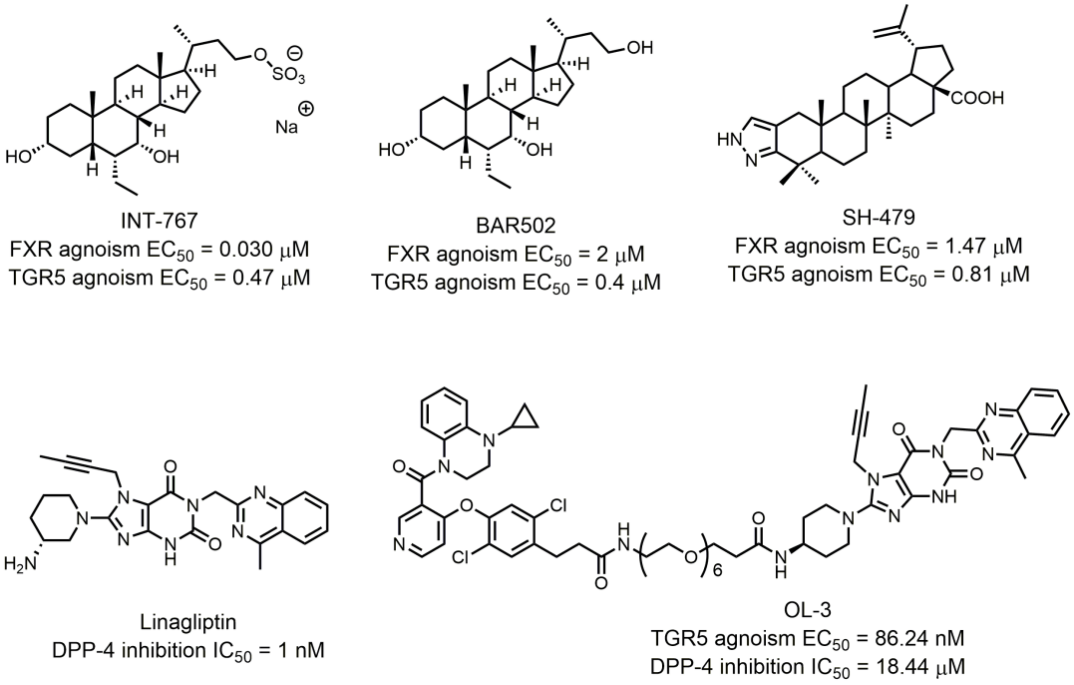
FIGURE 7: FXR and TGR5 dual targeting agonists and other dual targeting compounds.

Researchers found that chemical modulation of the side chain of steroidal cores? can change the selectivity of semisynthetic bile acid derivatives. Through introducing a sulfate group at the C-23 position of the 6α-ethylcholane scaffold, Rizzo *et al.* discovered INT-767, which was not only a very potent FXR agonist but also a potent activator of TGR5 (FXR agonism EC_50_ = 0.03 μM; TGR5 agonism EC_50_ = 0.47 μM; **Fig. 7**) [80]. INT-767 showed low cytotoxic effects and was highly stable to phase I and II enzymatic modifications, suggesting its potential clinical application in the treatment of liver and metabolic diseases as a safe and effective dual-targeting modulator. Currently, INT-767 is the most advanced compound in preclinical trials. BAR502 is another advanced compound that acts as a dual-targeting modulator of FXR/TGR5 (**Fig. 7**). In their systematic SAR studies, Festa *et al.* discovered that a hydroxyl group on the C-23 position of the 6α-ethylcholane scaffold generated dual-targeting agonist BAR502 (FXR agonism EC_50_ = 2 μM; TGR5 agonism EC_50_ = 0.4 μM) [54]. Although BAR502 was a preferential FXR ligand, its potency to TGR5 agonism was satisfactory as a dual-targeting modulator. Cipriani *et al.* demonstrated that BAR502 could attenuate liver damage in murine models of cholestasis without inducing itching, suggesting agonisms of both receptors [81]. As mentioned before, active triterpene, like Hedragonic acid and BTA, are another kind of molecules being able to activate FXR and TGR5, so it is possible that FXR/TGR5 dual-targeting agonists can be triterpene. Li *et al.* screened 35 BTA derivatives using TGR5-dependent cAMP accumulation and FXR response element reporter gene expression [79]. They found that SH-479, which was first synthesized by Xu *et al.* [82], was a potent TGR5 and FXR dual agonist (**Fig. 7**). SH-479 abrogated bone loss in a C57BL/6J mouse model through bone remodeling pathways, showing its potential as a therapeutic strategy for osteoporosis.

Dual modulators for other targets were also developed to treat metabolic diseases. Inspired by the molecular design of compound **16**, Ma *et al.* designed and synthesized OL-3, a novel low-absorbed TGR5 agonist (EC_50_ = 86.24 nM) with dipeptidyl peptidase-4 (DPP-4) inhibitory activity (IC_50_ = 18.44 μM; **Fig. 7**) [83]. Circulating GLP-1 has a short half-life due to the presence of DPP-4, so DPP-4 inhibitors can prevent the inactivation process of GLP-1, thus improving the level of active GLP-1 [84]. Linagliptin is one of the most selective and potent inhibitors of DPP-4 approved by the FDA in 2011 (IC_50_ = 1 nM; **Fig. 7**). OL-3 was generated by linking the structure of compound **15** and linagliptin with the PEG_6_ linker. It showed low cell permeability in Caco-2 cells and low absorption *in vivo.* Oral administration of OL-3 significantly lowered blood glucose levels but did not cause gallbladder filling, suggesting its high safety when being used.

## BILE ACID COMPOSITION AND CYP8B1

**Figure 8:**
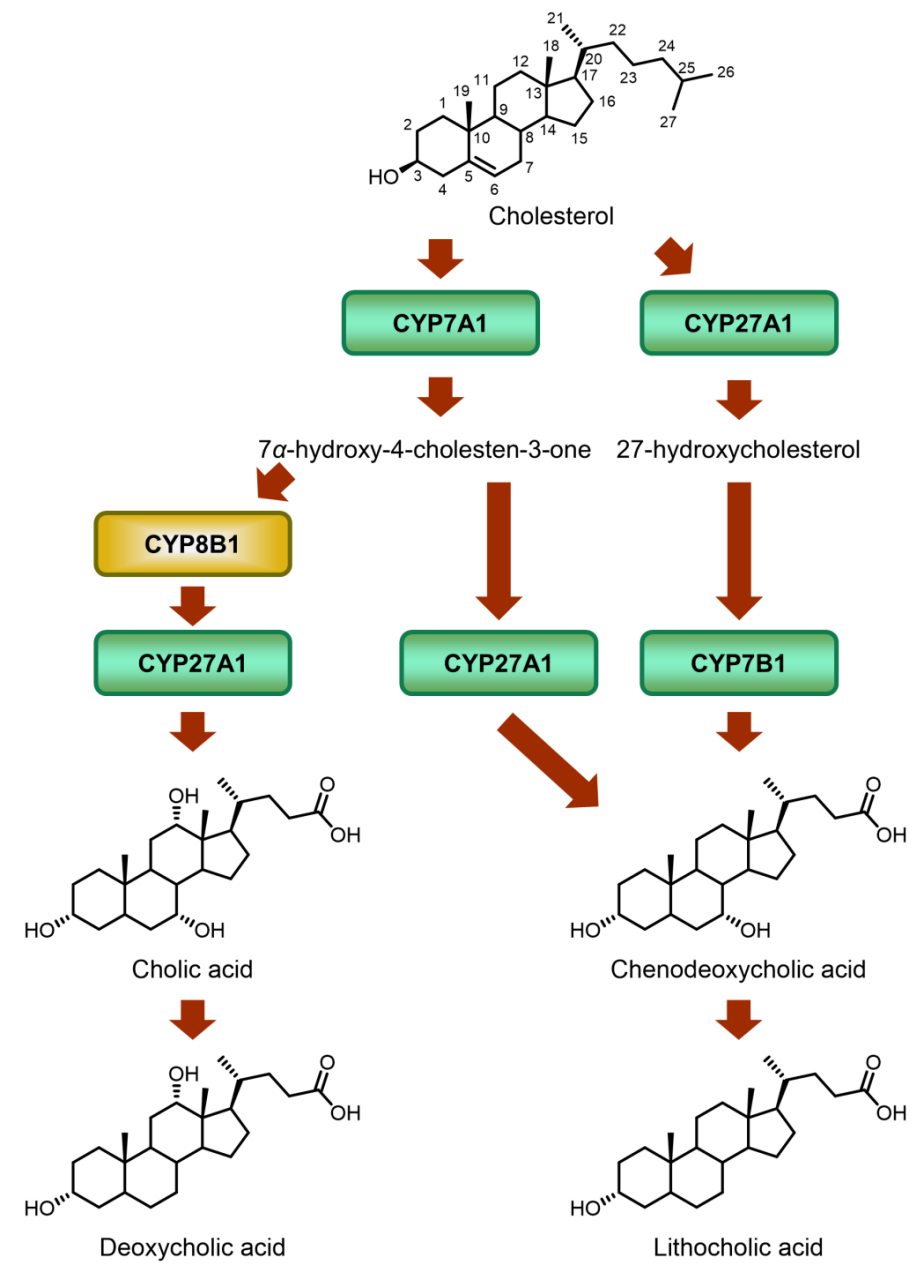
FIGURE 8: Biosynthesis of bile acids. Bile acids are synthesized in liver through two pathways: the classic pathway and the alternative pathway. CYP7A1 initializes the classic pathway, while CYP27A1 initializes the alternative pathway. CYP8B1 regulates the CA synthesis in the classic pathway, and is the only enzyme catalyzing the CA synthesis. In the intestine, primary bile acid CA and CDCA are metabolized by the gut microbiota to produce the secondary bile acids, DCA and LCA, respectively.

Bile acids are the general name of several steroid acids. In humans, the bile acid pool consists of CA, CDCA, Deoxycholic acid (DCA), LCA, and glycine (G)- or taurine (T)-conjugated bile acids [15]. Conversion of cholesterol to bile acids in the liver is the major pathway for catabolism of cholesterol (**Fig. 8**) [85]. Among those bile acids, CA and CDCA are synthesized in the liver, and are called primary bile acids. CA is more hydrophilic than CDCA, which means that CA can cause higher lipid absorption in the intestine [86]. Furthermore, CA and CDCA can act differently as hormones [87]. As a result, the ratio of CA and CDCA plays an important role in fat absorption and homeostasis. Sterol 12a-hydroxylase (CYP8B1) is the key enzyme for CA synthesis. Research indicates that inhibition of CYP8B1 in mice improves metabolism. Slatis *et al.* demonstrated that knocking out *Cyp8b1* in an *ApoE* knock out mouse model reduced atherosclerotic plaques [88]. Kaur *et al.* showed that glucose homeostasis in *Cyp8b1*^*-/-*^ mice could be improved with increasing GLP-1 [89]. Chevre *et al.* reported that in a cholesterol-induced NAFLD mouse model, inhibition of *Cyp8b1* expression led to a regression of hepatic steatosis [90]. These studies suggest that human CYP8B1 is a viable therapeutic target. Using a HFD-induced obese C57Bl/6 mouse model, Myronovych *et al.* found that *Cyp8b1* expression was downregulated after VSG [11]. Although VSG mice and sham operation mice achieved weight loss, the VSG mice had a significantly lower hepatic triglyceride content, which suggests that the improved metabolic outcomes were caused by more than just the early weight lost post-surgery. Developing CYP8B1 inhibitors is a promising way for treating glucose and lipid metabolic diseases at a lower cost and with reduced risk.

Unlike in humans, the majority of CDCA in mice is typically converted to muricholic acid (MCA) by CYP2c70 which exists in rodents but not humans [13, 91–94]. Therefore, in addition to the slump of CA level, the *Cyp8b1* knock out also results in a remarkable increase of MCA levels in mice [95]. Accordingly, the bile acid composition in either wild type or *Cyp8b1* knock out mice differs substantially from that in humans. However, both CDCA and MCA belong to non-12α-OH bile acids. Moreover, although MCA is a hydrophilic 6-hydroxylated bile acid, whereas CDCA is more hydrophobic, both have been reported to significantly lower cholesterol absorption as compared to CA [96]. Thus, despite those differences in bile acid composition, lipid absorption may be similarly affected in humans and mice upon CYP8B1 downregulation due to comparable changes in the 12α-OH/non-12α-OH BA ratio.

**Figure 9:**
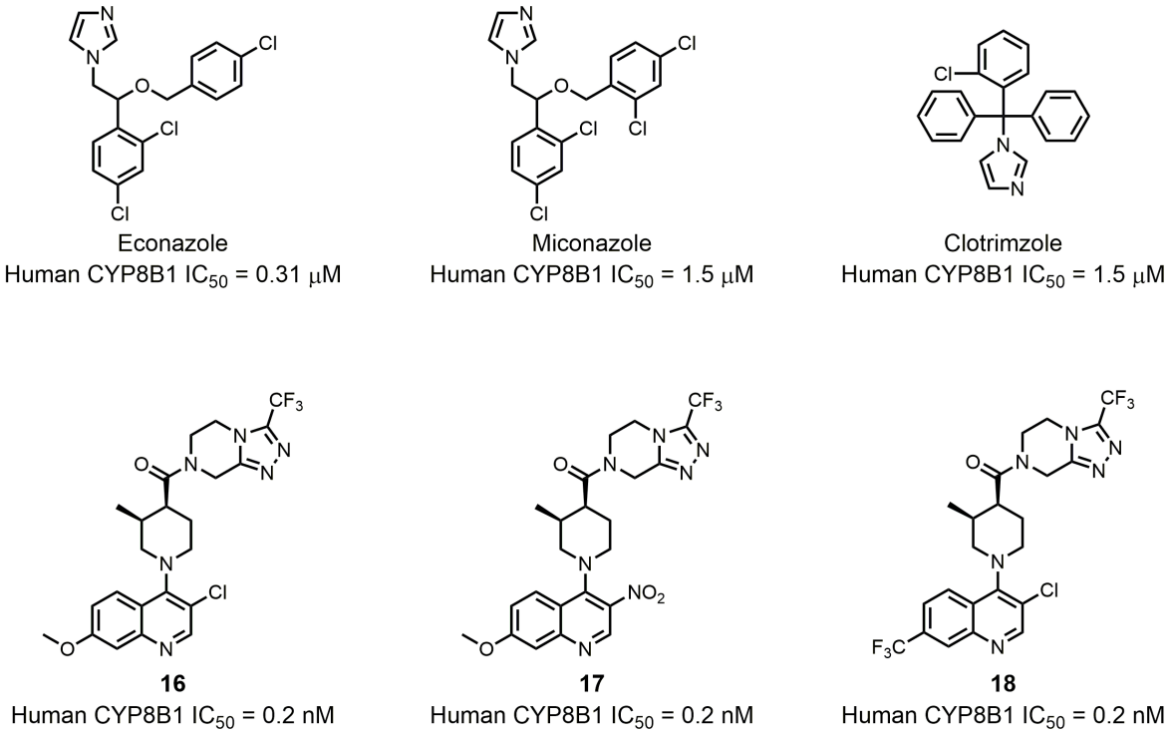
FIGURE 9: CYP8B1 inhibitors.

To date, several small molecule inhibitors of CYP8B1 have been reported, which laid a foundation for future drug design. Franchini *et al.* reported several agents showing CYP8B1 inhibitory activity [97]. Among them, econazole, clotrimazole and miconazole are potent inhibitors exhibiting IC_50_ < 2 μM (**Fig. 9**). Caldwell *et al.* reported a series of compounds showing strong CYP8B1 inhibitory activity, with all compounds having a similar core structure (**Fig. 9**) [98]. The most potent compound **16**, **17** and **18** exhibited IC_50_ = 0.2 nM. Fan *et al.* established a rapid and convenient CYP8B1 inhibitor test system, and tested six inhibitors of other CYPs using this system [99]. They successfully found ketoconazole, letrozole, exemestane and aminobenzotriazole as potential CYP8B1 inhibitors, which inhibited more than 10% of CYP8B1 at concentrations lower than 10 μM (**Table 1**). All these inhibitors have not been tested for their selectivity for CYP8B1. Developing inhibitors with high potency and selectivity is a highly challenging work. These compounds might serve as a starting point for further medicinal chemistry work.

**TABLE 1.** Effect of inhibitors on CYP8B1 activity.

**Compound**	**Structure**	**Percentage inhibition of CYP8B1**
Letrozole	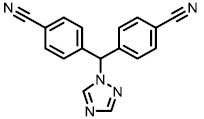	20 ± 5^d^
Exemestane	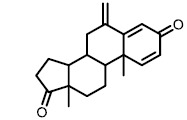	17 ± 11^c^
Ketoconazole	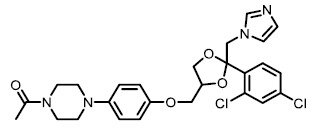	10 ± 8^b^
Aminobenzotriazole	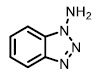	16 ± 10^c^

a Inhibitor concentration: 10 μM. Significant differences:

b p < 0.01

c p < 0.005

d p< 0.0001.

## GUT MICROBIOME AND BILE SALT HYDROLASE

Intestinal microbiota disorders can lead to a variety of diseases, such as obesity and colon cancer, implicating the importance of microbes in the human gut in maintaining healthy metabolism [100]. The influence on metabolism brought by intestinal microbiota can be explained by the bidirectional relationship between the bile acids and the gut microbial populations [101]. Different therapeutic strategies have been developed based on the understanding of this relationship.

**Figure 10:**
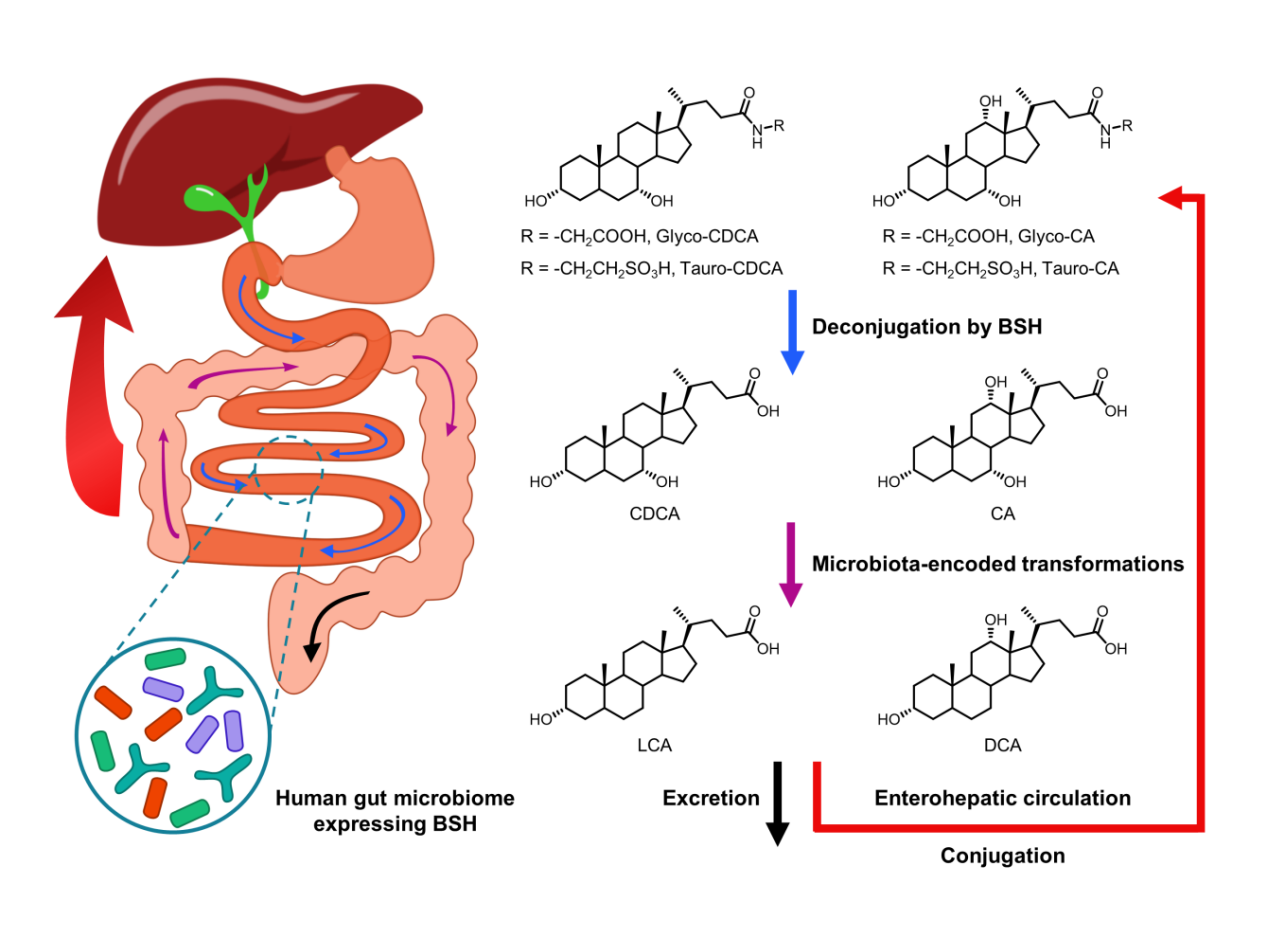
FIGURE 10: Biotransformation of bile acids by BSH-containing gut microbiota.

Bile acids synthesized in the liver can regulate intestinal microbiota through a direct or indirect effect. Conjugated bile acids directly alter bacterial membrane architecture and damage DNA, which inhibit growth of bacteria [102, 103]. Bile acids can also indirectly affect microbiota via nuclear receptors. Inagaki *et al.* showed that FXR activation by bile acids can induce the expression of genes that inhibit microbial overgrowth and mucosal damage [104]. These results are consistent with the idea that bile acids are integral in controlling intestinal microbiota, and therefore has significant implications for maintaining equilibrium between human host and intestinal microbiota. According to a systematic study that profiled gut microbiota-related metabolites, Liu *et al.* found higher serum glutamate concentrations and reduced abundance of *Bacteroides thetaiotaomicron* in overweight and obese Chinese individuals [105]. Following weight-loss intervention by VSG, analysis of these metabolomes in 23 obese individuals showed a return to baseline levels of both *B. thetaiotaomicron* and the serum glutamate concentrations. Moreover, gavage of live *B. thetaiotaomicron* in mice decreased levels of glutamate concentration and alleviated the diet-induced body-weight gain. The alterations in the population of *B. thetaiotaomicron* in obese individuals after bariatric surgery were possibly caused by the modifications of the bile acid pool. This result highlights the potential for obesity interventions by targeting the gut microbiota.

**Figure 11:**
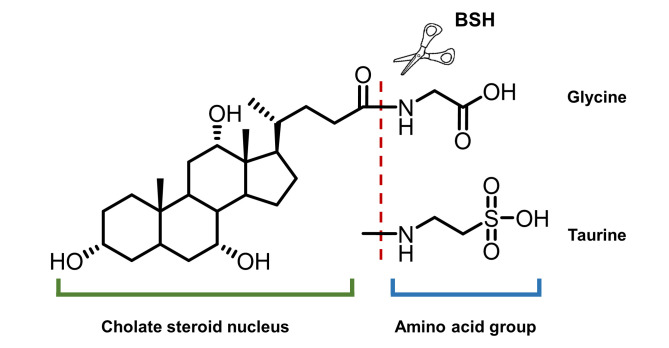
FIGURE 11: General structures of bile salts and the functions of BSHs.

On the other hand, intestinal microbiota can change the size and composition of the bile acid pool via biotransformation (**Fig. 10**). Bile salt hydrolase (BSH) is an enzyme catalyzing the hydrolysis of glycine- and/or taurine-conjugated bile acids into unconjugated bile acids and amino acid residues (**Fig. 11**) [106]. Conjugated bile acids consist of a hydrophobic steroid core and a hydrophilic amino acid side chain (glycine or taurine). The hydrophilicity of amino acid chains render conjugated bile acids to be better biological detergents than unconjugated bile acid because of their ability to emulsify dietary lipids for fat digestion in the small intestine [107]. For this reason, deconjugation of bile acids by BSHs may induce weight loss in the setting of malabsorption [108]. Inversely, intestinal BSH inhibition may be helpful for energy harvest. In fact, many efforts have been made to develop BSH inhibitors as novel non-antibiotic growth promoters to enhance animal production and health [109]. It is highly possible that small molecule BSH agonists can decrease weight gain in obese patients through the mechanism of malabsorption. However, no compound has been reported that can mediate such an effect. Fortunately, prebiotics or probiotics have been reported to improve glucose and lipid metabolism [110].

**Figure 12:**
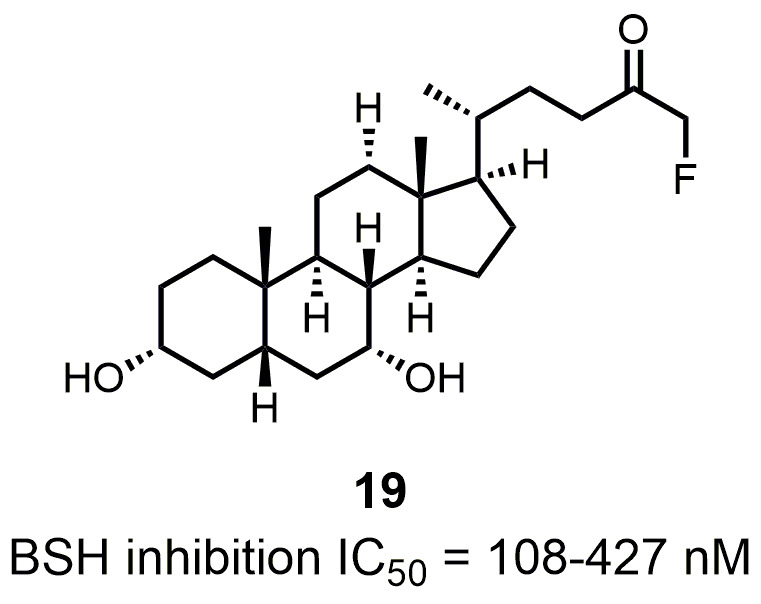
FIGURE 12: Structure and activity of the covalent pan-inhibitor of BSHs.

The detergent nature of bile acids creates reason to believe that inhibition of BSH can reduce host weight gain. However, different bile acids can act differently in human bodies as hormones. Under this condition, the relationship between BSH activity and metabolism of the host might change. Yao *et al.* reported that in gnotobiotic mice mono-colonized with wild type and BSH-deleted *B. thetaiotaomicron*, the BSH-deleted mice demonstrated significant reduction in weight gain [111]. In mice, the two most abundant murine-conjugated bile acids found were tauro-β-muricholic acid (TβMCA) and tauro-cholic acid (TCA). BSH from *B. thetaiotaomicron* was able to selectively deconjugate TβMCA, but not TCA. This result demonstrated that inhibition of selective BSHs might benefit the host metabolism. For this reason, development of BSH inhibitors is underway. Wang *et al.* found that copper (CuCl_2_) and zinc (ZnSO_4_) could be potent BSH inhibitors, and they were able to promote food digestion and body weight gain in different animal models [112]. Smith *et al.* identified three BSH inhibitors (CAPE, Riboflavin and Carnosic Acid) using a high-throughput screening method with a recombinant BSH, which contains broad substrate specificity from a chicken *Lactobacillus salivarius*, the dominant lactic acid bacterium present in the chicken intestine (**Table 2**) [113]. However, these inhibitors showed either no inhibitory or moderate inhibitory activity to other BSHs like *B. thetaiotaomicron* and *Bifidobacterium longum* BSHs, which largely inhibit their clinical use in humans [114]. Although BSH protein sequences vary largely between different gut strains, all BSHs possess a conserved active site that includes a catalytic cysteine (Cys2) [115]. Based on this property, Adhikari *et al.* developed a covalent inhibitor, compound **19** (**Fig. 12**), targeting both *B. thetaiotaomicron* and *B. longum* BSHs in a dose-dependent fashion (IC_50_ = 108-427 nM) [114]. Compound **19** could effectively inhibit deconjugation of bile salts *in vitro* and *in vivo*, but did not significantly affect the viability of gut bacteria. This research is meaningful for the investigation of how BSH activity directly affects metabolism in fully colonized hosts, which is more likely to mimic the real situation. Other pan-inhibitors and selective inhibitors of BSHs, which might beneficially affect host physiology, could be developed based on this work.

## PERSPECTIVES

Among the bile acid related targets discussed above, FXR and TGR5 lead the way in the drug development pipeline. Since FXR and TGR5 may play different roles in different metabolic diseases, it is possible to design small molecules with various potency and selectivity depending on patient profiles. The metabolic role of CYP8B1 has been recently explored; thus, CYP8B1 inhibition may offer a promising therapeutic avenue for treating metabolic diseases. Currently, a few small molecules have been reported to have CYP8B1 inhibitory activity. However, they suffer from low potency and selectivity. More medicinal chemistry studies are required for the development of new CYP8B1 inhibitors. The role of BSHs in metabolism is more dynamic and complex; it is not clear whether BSH agonism or inhibition can be beneficial to the host metabolism. Further studies are warranted to better understand the role of BSHs in regulating metabolism to create an opportunity for drug discovery. On the other hand, through the use of prebiotics, probiotics, and gene editing technologies, more studies can be conducted to improve the overall understanding of BSHs as drug targets.

**TABLE 2.** Effect of inhibitors on BSH activity ^a^.

**Compound**	**Structure**	**Percentage inhibition of BSH**
CAPE	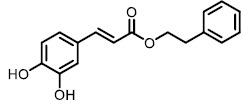	99.7
Riboflavin^b^	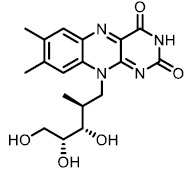	99.3
Carnosic Acid	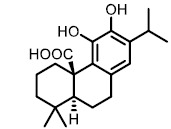	96.8

a Unless specified, the final concentration of compound in the reaction mix was 5 mM to achieve optimal resolution with the quantitative BSH activity assay.

b The final concentration of riboflavin in reaction mix was 1 mM.

## Abbreviatons:

6-ECDCA: – 6α-ethyl-chenodeoxycholic acid;
BSH: – bile salt hydrolase;
BTA: – betulinic acid;
CA: – cholic acid;
CDCA: – chenodesoxycholic acid;
DHA: – dihydroartemisinin;
DPP-4: – dipeptidyl peptidase-4;
FXR: – farnesoid X receptor;
GLP-1: – glucagon-like peptide-1;
HFD: – high-fat diet;
LCA: – lithocholic acid;
MCA: – muricholic acid;
miR-26a: – microRNA-26a;
NAFLD: – nonalcoholic fatty liver disease;
NASH: – nonalcoholic steatohepatitis;
OA: – oleanolic acid;
PBC: – primary biliary cholangitis;
PEG: – polyethylene glycol;
PYY: – peptide YY;
RYGB: – Roux-en-Y gastric bypass;
SAR: – structure-activity relationship;
TβMCA: – tauro-β-muricholic acid;
TCA: – tauro-cholic acid;
TGR5: – Takeda G-protein-coupled receptor 5;
TLCA: – taurolithocholic acid;
UDCA: – ursodeoxycholic acid;
VSG: – vertical sleeve gastrectomy.
